# Overlapping conditions in Long COVID at a multisite academic center

**DOI:** 10.3389/fneur.2024.1482917

**Published:** 2024-10-25

**Authors:** Stephanie L. Grach, Daniel V. Dudenkov, Beth Pollack, DeLisa Fairweather, Chris A. Aakre, Bala Munipalli, Ivana T. Croghan, Michael R. Mueller, Joshua D. Overgaard, Katelyn A. Bruno, Nerissa M. Collins, Zhuo Li, Ryan T. Hurt, Michal C. Tal, Ravindra Ganesh, Dacre T. R. Knight

**Affiliations:** ^1^Division of General Internal Medicine, Mayo Clinic, Rochester, MN, United States; ^2^Department of General Internal Medicine, Mayo Clinic, Jacksonville, FL, United States; ^3^Department of Biological Engineering, Massachusetts Institute of Technology, Cambridge, MA, United States; ^4^Department of Cardiovascular Medicine, Mayo Clinic, Jacksonville, FL, United States; ^5^Center for Clinical and Translational Science, Mayo Clinic, Rochester, MN, United States; ^6^Department of Immunology, Mayo Clinic, Jacksonville, FL, United States; ^7^Division of Quantitative Health Sciences, Rochester, MN, United States; ^8^Department of Cardiovascular Medicine, University of Florida, Gainesville, FL, United States; ^9^Department of Biostatistics, Mayo Clinic, Jacksonville, FL, United States; ^10^Institute for Stem Cell Biology & Regenerative Medicine, Stanford University School of Medicine, Stanford, CA, United States

**Keywords:** SARS-CoV-2, generalized joint hypermobility, myalgic encephalomyelitis/chronic fatigue syndrome, hypermobile Ehlers-Danlos syndrome, orthostatic intolerance, pain, virus, mast cells

## Abstract

**Background:**

Many patients experience persistent symptoms after COVID-19, a syndrome referred to as Long COVID (LC). The goal of this study was to identify novel new or worsening comorbidities self-reported in patients with LC.

**Methods:**

Patients diagnosed with LC (*n* = 732) at the Mayo Long COVID Care Clinic in Rochester, Minnesota and Jacksonville, Florida were sent questionnaires to assess the development of new or worsening comorbidities following COVID-19 compared to patients with SARS-CoV-2 that did not develop LC (controls). Both groups were also asked questions screening for myalgic encephalomyelitis/chronic fatigue syndrome (ME/CFS), generalized joint hypermobility (GJH) and orthostatic intolerance. 247 people with LC (33.7%) and 40 controls (50%) responded to the surveys.

**Results:**

In this study LC patients averaged 53 years of age and were predominantly White (95%) women (75%). The greatest prevalence of new or worsening comorbidities following SARS-CoV-2 infection in patients with LC vs. controls reported in this study were pain (94.4% vs. 0%, *p* < 0.001), neurological (92.4% vs. 15.4%, *p* < 0.001), sleep (82.8% vs. 5.3%, *p* < 0.001), skin (69.8% vs. 0%, *p* < 0.001), and genitourinary (60.6% vs. 25.0%, *p* = 0.029) issues. 58% of LC patients screened positive for ME/CFS vs. 0% of controls (*p* < 0.001), 27% positive for GJH compared to 10% of controls (*p* = 0.026), and a positive average score of 4.0 on orthostatic intolerance vs. 0 (*p* < 0.001). The majority of LC patients with ME/CFS were women (77%).

**Conclusion:**

We found that comorbidities across 12 surveyed categories were increased in patients following SARS-CoV-2 infection. Our data also support the overlap of LC with ME/CFS, GJH, and orthostatic intolerance. We discuss the pathophysiologic, research, and clinical implications of identifying these conditions with LC.

## 1 Introduction

Soon after the start of the COVID-19 pandemic, patients presented to clinicians with new or worsening symptoms that persisted for many months after the initial SARS-CoV-2 infection (1, 2). This syndrome has been named Long COVID (LC), Post-COVID syndrome, and Post-Acute Sequelae of SARS-CoV-2 Infection (PASC). The US Centers for Disease Control and Prevention (CDC) and the US Department of Health and Human Services define LC as signs, symptoms, and conditions persisting for at least 4 weeks following COVID-19 (3). The National Academies of Science, Engineering, and Medicine (NASEM) defines LC as an infection-associated chronic condition following SARS-CoV-2 infection that lasts at least 3 months, affects at least one organ system, and can be continuous, relapsing, or remitting (4). In the first quarter of 2024, approximately 5.3% of US adults currently were estimated to have LC by the CDC (5). Consequently, the projected economic impact ranges from $140 to $600 billion annually (6).

LC impacts multiple organ systems, leading to exertional intolerance, fatigue, dyspnea, muscle and joint pain, orthostatic intolerance, and cognitive dysfunction (4, 7–9). An electronic health record (EHR) study of over 20,000 people with LC in Florida used an artificial intelligence program to identify subgroups of patients with different organ systems affected by LC that included cardiac and renal (34%); respiratory, sleep, and anxiety (33%); musculoskeletal and nervous (23%); and digestive and respiratory (10%) systems (10). LC is also more likely to occur in patients who are middle-age and of female sex (1, 11–13). CDC assessments estimate that LC also has a disproportionate impact on the transgender, Hispanic, and multiracial populations (5).

LC stands as a prominent recent example of how an infectious agent can be associated with chronic disease (14–17). Infectious disease is an established cause of neurodegeneration, including neurotropic infections such as Herpesviridae, Bornaviridae, Orthomyxoviridae (including influenza), Paramyxoviridae, Picornaviridae, Retroviridae and Flaviviridae (18–20). Post-infectious onset and the role of latent viruses is also increasingly appreciated in the development of myalgic encephalomyelitis/chronic fatigue syndrome (ME/CFS) among other related conditions (21–25). Accordingly, LC research is quickly revealing high rates of multisystem complex chronic diseases such as ME/CFS and postural orthostatic tachcyardiac syndrome (POTS) (26–30). Notably, these conditions are highly comorbid with each other, along with generalized joint hypermobility (GJH), hypermobile Ehlers-Danlos Syndrome (hEDS), and hypermobility spectrum disorders (HSD) (31–36). The constellation of symptoms and comorbidities reported in LC seem to be very similar to those found in ME/CFS, orthostatic intolerance/POTS, and hEDS/HSD (31–38).

Little data exists on a wide range of comorbidities that may arise with LC. The goal of this study was to determine whether new or worsening conditions occurred more frequently in patients diagnosed with LC at our clinic compared to COVID patients that did not develop LC. We additionally examined whether patients with LC were more likely to screen positive for ME/CFS, GJH, or orthostatic intolerance. We propose that the identification of overlapping conditions and their potential underlying mechanisms may offer important insight into research and clinical management of LC.

## 2 Methods

### 2.1 Diagnoses

Individuals attending the Long COVID Care Clinic at Mayo Clinic Florida and Rochester were diagnosed by general internal medicine physicians specialized in LC. A majority of LC physicians had additional specialization in fibromyalgia, ME/CFS, POTS, and/or hypermobility syndromes (13). All patients diagnosed with LC had persistent symptoms for at least 3 months following the start of acute SARS-CoV-2 infection. Controls had a confirmed history of SARS-CoV-2 infection but had not received a diagnosis of LC.

### 2.2 Long COVID clinic data collection

Patient data were collected over 1 month using two REDCap questionnaires sent to patients via the patient portal. The questionnaires were based on the Florida EDS Clinic questionnaire for patients with hEDS/HSD, with questions added or modified for LC, ME/CFS, POTS, and other conditions increasingly suspected in infection-associated chronic conditions (39, 40). Dedicated screening criteria for hypermobility, ME/CFS, and orthostatic intolerance were added. The Long COVID questionnaire obtained self-reported data on 115 demographic and health issues in total. This was sent to a sample of patients diagnosed with LC at the Mayo Clinic Long COVID Clinic in Jacksonville, Florida and Rochester, Minnesota. Health information focused on symptoms and comorbidities throughout the body and whether specific comorbidities were new or worse following COVID. A matching control questionnaire was created where wording was modified to ask participants about symptoms since prior COVID infections without mention of Post-COVID Syndrome or Long COVID, and was sent to a control group in Rochester, Minnesota consisting of individuals who had had prior COVID infection without diagnosis of LC. Specifically, the control participants were from a developing age-sex matched cohort specifically for Long COVID studies, currently in process of recruiting with 80 subjects enrolled at the time of the survey. The Long COVID questionnaire was sent to 494 patients in Florida and 238 patients in Minnesota. A total of 247 individuals completed the Long COVID questionnaire (33.7% response rate): 124 (25.1% response rate, 50.2% of total responses) from Florida and 123 (51.7% response rate, 49.8% of total responses) from Minnesota. The control questionnaire was completed by 40 individuals from Minnesota (50% response rate).

### 2.3 Statistical analysis

Categorical variables were summarized as frequency (percentage) and continuous variables were reported as median (range). Wilcoxon rank sum test was used to evaluate the difference in continuous variables between LC patients and controls while Fisher’s exact test was used to compare categorical variables. Multivariable logistic regression models were used to evaluate the difference between LC and control patients after adjusting for age and race (White vs. non-White). Firth’s penalized logistic regression was used for outcomes with zero events in the control group. All tests were two-sided and *p* values <0.05 were considered statistically significant. The analysis was performed using R (version 4.2.2).

### 2.4 Categories

We assessed whether patients diagnosed with LC observed new or worsening conditions for 14 major systems categories after SARS-CoV-2 infection compared to controls: allergies/sensitivities/intolerances, skin, neurologic, pain, sleep, autoimmune, genitourinary, mood, gastrointestinal, pulmonary, cardiac, endocrinologic, hematologic/oncologic, and dental disorders, as included in the original questionnaire.

A portion of the major categories also included an expanded set of specific conditions that respondents could select based on expert opinion of potentially common and/or underrecognized coexisting conditions (39). Those categories and conditions included:

- Skin: easy bruising, poor/slow wound healing, easy/abnormal scarring, abnormal striae, lipedema, psoriasis, eczema, rosacea, hives, and flushing.
- Neurologic: headache, migraine, Arnold Chiari malformation, intracranial hypertension/elevated CSF pressure, CSF leak, autonomic dysfunction not meeting criteria for postural orthostatic tachycardia syndrome, postural orthostatic tachycardia syndrome, vertigo, neuropathy (small and/or large), tinnitus, craniocervical instability, myalgic encephalomyelitis/chronic fatigue syndrome, autism/autism spectrum disorder, inappropriate sinus tachycardia, and recurrent syncope.
- Pain: fibromyalgia, chronic pain—non-muscular, chronic muscle pain, cervicalgia, central pain syndrome, central sensitization, muscle spasms, and complex regional pain syndrome.
- Sleep: obstructive sleep apnea, snoring, narcolepsy, idiopathic hypersomnia, insomnia, sleep disturbance, restless leg syndrome, parasomnia, and circadian rhythm disorders (such as delayed sleep phase syndrome).
- Genitourinary: bladder prolapse, uterine prolapse, recurrent urinary tract infections, recurrent yeast infections, recurrent vaginal bacterial infections, incontinence, pelvic floor spasm, pelvic floor dysfunction, dyspareunia, interstitial cystitis, endometriosis, and polycystic ovary syndrome (PCOS). Sexual and menstrual concerns were surveyed separately.

Finally, a miscellaneous category was included for emerging phenomena within infection-associated chronic illness to assess the level of recognition of those comorbidities, including mast cell disease, endothelial dysfunction, viral persistence/reactivation, vascular compression syndromes, mitochondrial impairment, and sensitivity to medications, based on expert discussion surrounding emerging areas in the field of infection-associated chronic conditions.

### 2.5 Screening for hypermobility, ME/CFS, orthostatic intolerance

Several screening criteria were embedded in the REDCap questionnaires to determine whether patients had ME/CFS, GJH, or orthostatic intolerance. The Institute of Medicine/National Academy of Science, Engineering and Medicine (IOM/NASEM) 2015 criteria were used for ME/CFS (41). The self-assessment 5-part hypermobility questionnaire was utilized to assess GJH. This is used in clinical practice to screen for hEDS/HSD as well, acknowledging that GJH may not always be symptomatic and is not alone sufficient to obtain a diagnosis of hEDS or HSD, where other criteria are required (42–44). A modified version of the orthostatic intolerance domain of the Composite Autonomic Symptom Scale 31 (COMPASS-31) questionnaire was used for orthostatic intolerance screening (45) (Supplementary material 1).

## 3 Results

### 3.1 Patient demographics

Controls were older (58.5 years) than patients with LC (53.0 years) (*p* = 0.021) (Table 1). However, most controls (70.0%) and patients with LC (75.3%) self-reported being female, which was not significantly different between groups (*p* = 0.52) (Table 1). Controls (100%) and patients with LC (93.9%) self-reported as White vs. non-White for race (*p* = 0.24) (Table 1).

**Table 1:** Demographics.

	Control (*n* = 40) (*n*, %)	Long COVID (*n* = 247) (*n*, %)	*p* value
Age, median (range)	58.5 (28.0, 83.0)	53.0 (18.0, 84.0)	0.021
Sex			0.520
Female	28 (70.0%)	186 (75.3%)	
Male	12 (30.0%)	60 (24.3%)	
Not disclosed	0 (0.0%)	1 (0.4%)	
LGBTQIA^a^	3 (7.5%)	19 (7.7%)	
Race			0.240
White	40 (100.0%)	232 (93.9%)	
Not White	0 (0.0%)	15 (6.1%)	

a LGBTQIA; lesbian, gay, bisexual, transgender, queer, intersex or asexual.

### 3.2 New or worsening comorbidities after COVID

New or worsening conditions were reported in 11 major systems categories, as well as in the miscellaneous category (Table 2). The major systems included, in descending order of frequency, pain conditions (94.4% vs. 0%, *p* < 0.001), neurological conditions (92.4% vs. 15.4%, *p* < 0.001), sleep issues (82.8% vs. 5.3%, *p* < 0.001), skin issues (69.8% vs. 0%, *p* < 0.001), genitourinary issues (60.6% vs. 25.0%, *p* = 0.029), allergies/sensitivities/intolerances (31.8% vs. 10.0%, *p* < 0.001), mood disorders (31.6% vs. 7.5%, *p* = 0.001), gastrointestinal disorders (21.9% vs. 2.5%, *p* = 0.002), autoimmune issues (16.2% vs. 0%, *p* = 0.006), pulmonary disorders (15.8% vs. 0%, *p* = 0.002), and cardiac disorders (12.1% vs. 0%, *p* = 0.012) (Table 2). Presence of a new or worsening comorbidity following COVID-19 in LC patients that were found to be significant are shown in Table 3, adjusted for age and race. Female sex was associated with increased risk of neurologic manifestations and hypermobility in LC (Table 4; Supplementary material 2).

**Table 2:** New or worsening comorbidities after COVID-19.

	Control (*n* = 40)	Long COVID (*n* = 247)	*p* value
Did your **pain** condition(s) start or worsen after COVID?	0 (0.0%)	169 (94.4%)	<0.001
Did any of your **neurologic** condition(s) start or worsen after COVID?	2 (15.4%)	195 (92.4%)	<0.001
Did your **sleep** condition(s) start or worsen after COVID?	1 (5.3%)	144 (82.8%)	<0.001
Did your **skin** condition(s) start or worsen after COVID?	0 (0.0%)	90 (69.8%)	<0.001
Did at least one **genitourinary** condition start or worsen after COVID?	3 (25.0%)	66 (60.6%)	0.029
**Allergies**, sensitivities/intolerances			<0.001
*Allergies, sensitivities/intolerances stayed the same	21 (52.5%)	68 (28.1%)	
*Allergies, sensitivities/intolerances new after COVID	4 (10.0%)	77 (31.8%)	
*Allergies, sensitivities/intolerances before COVID got worse	3 (7.5%)	55 (22.7%)	
Diagnosis of a **mood** disorder (e.g., depression, anxiety) after COVID	3 (7.5%)	78 (31.6%)	0.001
Diagnosis of a **gastrointestinal** disorder (e.g., inflammatory bowel syndrome) after COVID	1 (2.5%)	54 (21.9%)	0.002
Have you been diagnosed with an **autoimmune** disorder?			0.006
*Before COVID	4 (10.0%)	35 (15.0%)	
*After COVID	0 (0.0%)	38 (16.2%)	
*Before + more after COVID	0 (0.0%)	5 (2.1%)	
Diagnosis of a **pulmonary** disorder (e.g., asthma, interstitial lung disease) after COVID	0 (0.0%)	39 (15.8%)	0.002
Diagnosis of a **cardiac** disorder (e.g., myocarditis, heart failure) after COVID	0 (0.0%)	30 (12.1%)	0.012

**Table 3:** Multivariable logistic regression model evaluating new or worsening comorbidities after COVID between Long COVID and controls, adjusted for age and race (White).

Outcome	OR (95% CI)	*p* value
Allergy	5.09 (2.26, 13.06)	<0.001
Sleep	138.31 (25.61, 2,585.15)	<0.001
Genitourinary	4.28 (1.13, 21.04)	0.04
Autoimmune	16.71 (2.28, 2,130.38)	0.001
Gastrointestinal	10.6 (2.19, 190.93)	0.02
Mood	5.42 (1.87, 23.06)	0.006
Cardiac	11.84 (1.6, 1,512.55)	0.009
Pulmonary	15.02 (2.04, 1,915.54)	0.003

**Table 4:** Assessment of generalized hypermobility, ME/CFS, and orthostatic intolerance.

	Control (*n* = 40) (*n*, %)	Long COVID (*n* = 247) (*n*, %)	*p* value
ME/CFS	0 (0.0%)	144 (58.3%)	<0.001
Females	0 (0.0%)	111 (77.1%)	
Males	0 (0.0%)	32 (53.3%)	
Hypermobility (GJH)^a^	4 (10.3%)	63 (27.0%)	0.026
Females	4 (100.0%)	57 (90.5%)	
Males	0 (0.0%)	6 (9.5%)	
Orthostatic intolerance	0.0 (0.0, 5.0)	4.0 (0.0, 7.0)	<0.001
Females	0.0 (0.0, 5.0)	4.0 (0.0, 7.0)	
Males	0.0 (0.0, 5.0)	4.0 (0.0, 7.0)	

a GJH, generalized joint hypermobility; ME/CFS, myalgic encephalomyelitis/chronic fatigue syndrome.

Within the miscellaneous category, participants were asked if conditions had been diagnosed or suspected by a clinician after COVID, with answer choices of yes, unknown/unsure, or no. Results of significance in LC vs. controls included mast cell issues (16.2% yes, 36.3% unsure vs. 2.4% yes, 5 unsure, *p* < 0.001), endothelial dysfunction (3.9% yes, 32.9% unsure vs. 0% yes or unsure, *p* < 0.001), viral reactivation (e.g., Epstein–Barr virus, herpesvirus, or other viruses) (18.7% yes, 17.8% unsure vs. 2.6% yes, 2.6% unsure, *p* < 0.001), vascular compression syndromes (3.6% yes, 8.5% unsure vs. 0% yes, 2.4% unsure, *p* = 0.039), and mitochondrial disease/dysfunction (5.2% yes, 23.9% unsure vs. 0% yes, 2.4% unsure, *p* < 0.001). The questionnaires also asked about sensitivity or resistance to medications. LC respondents were more likely to report sensitivity to medications than controls (22.5% vs. 4.9%, *p* = 0.007).

New or worsening diagnoses within the categories of dental, hematologic/oncologic, or endocrinologic disorders did not meet statistical significance in our survey.

### 3.3 Screened comorbidities

We screened patients for ME/CFS, GJH, and orthostatic intolerance. A majority of patients with LC reported ME/CFS symptoms (58.3%) compared to controls (0%) (*p* < 0.001) (Table 4). A similar proportion of patients with co-occurring LC and ME/CFS were female (59.7%) vs. male (53.3%) (*p* = 0.45) We found that around 27% of patients with LC reported GJH compared to only 10% of controls (*p* = 0.026) (Table 4). Most of the patients with co-occurring LC and GJH were female (90.5%) vs. male (9.5%) (*p* < 0.0001), and all controls with a positive screen were female (Table 4). LC patients reported orthostatic intolerance symptoms with a score of 4.0 after COVID compared to a score of 0 for controls (*p* < 0.001) (Table 2). Both males and females with LC had a score of 4.0 for orthostatic intolerance symptoms (*p* = 0.19).

## 4 Discussion

Our research demonstrates statistically significant reporting of new diagnoses or worsening of existing conditions following COVID-19 infection present across almost every organ system surveyed in LC compared to controls. Of the major systems and comorbidities that we examined in this study, ones that were new or worse in patients with LC vs. COVID controls were pain (94.4), neurological (92.4%), sleep (82.8%), skin (69.8%), genitourinary (60.6%), allergies/sensitivities/intolerances (31.8%), mood disorders (31.6%), sensitivity to medications (22.5%), gastrointestinal disorders (21.9%), viral reactivation (e.g., Epstein–Barr virus, herpesvirus, or other viruses) (18.7%), autoimmune diagnoses (16.2%), mast cell issues (16.2%), pulmonary disorders (15.8%), cardiac disorders (12.1%), and vascular compression syndromes (3.6%).

Notably, LC patients were more likely to screen positive for ME/CFS, orthostatic intolerance, or GJH than to report being formally diagnosed with one of these conditions. Almost 60% of our patients diagnosed with LC also met IOM/NAM ME/CFS 2015 criteria (41). This finding is consistent with other studies that reported 43–58% of patients with LC have ME/CFS (26–28). We also found that around 30% of LC patients had a positive screen for GJH according to the self-assessment 5-questions for hypermobility (42).

### 4.1 Comorbidities in LC and related conditions

Our study supports much of the current literature surrounding different comorbidities in LC, ME/CFS, orthostatic intolerance, hypermobile conditions, and related complex chronic disease. Having found that 30% of our LC respondents screened positive for GJH, our findings correspond to a recent UK study of 2,854 patients that also assessed GJH using the self-assessment 5-part hypermobility questionnaire that found that GJH was associated with a 30% increased risk of developing LC (46).

The most frequently reported symptoms in this study were pain and neurologic symptoms/conditions. The development of fatigue, chronic widespread pain, and muscle/joint pain are all common to LC, ME/CFS, fibromyalgia and hEDS/HSD (30, 47–49). New onset or worsening chronic pain has a prevalence of 45–70% across multiple studies of LC patients (50). A cross-sectional survey study of adults with LC in Bangladesh (*n* = 563) identified five types of commonly occurring pain in LC: muscle pain, chest pain, joint pain, headache, and abdominal pain, all of which were associated with a reduction in quality of life (51). Pre-existing chronic pain conditions are also risk factors for developing LC. In a retrospective EHR study of people with acute COVID (*n* = 1,038,402), having a chronic pain condition such as fibromyalgia, endometriosis, or irritable bowel syndrome increased the risk of developing LC by 1.47 (95% CI = 1.46, 1.47) (52). Our team also recently found that around 70% of patients seen at the Mayo Florida EDS Clinic are diagnosed with fibromyalgia, indicating high overlap between hEDS, HSD and fibromyalgia (39).

Multiple studies have found that a high percentage of LC patients also have ME/CFS (26–30). Craniocervical disease, Arnold Chiari malformations, idiopathic intracranial hypertension, and other spinal structural and flow issues have been associated with ME/CFS, fibromyalgia, and connective tissue disorders previously (35, 53–57). Additionally, an estimated 50–81% of people with ME/CFS have hypermobility (35, 58). Joint hypermobility disorders themselves are strongly associated with autonomic dysfunction, including chronic orthostatic intolerance and POTS in up to 48% of patients with hEDS/HSD, as well as headaches of various origin (59–64). Neurodivergent conditions including ADHD and autism also have a higher prevalence in people with joint hypermobility (43, 65, 66).

Orthostatic intolerance was frequent both with self-reporting and screening questions in LC. Previous studies have found that around 30–80% of LC patients fulfill the criteria for POTS (29, 67). Patients with LC can also have evidence of autonomic dysfunction including reduced cerebral blood flow and symptoms of orthostatic intolerance on a tilt table test without meeting the heart rate criteria for POTS (68). GJH, hEDS and HSD are strongly associated with autonomic dysfunction, with chronic orthostatic intolerance and POTS found in up to 48% of patients with hEDS/HSD (59–64).

Multiple studies have also found that sleep dysfunction is common in LC patients. A survey study of adults with LC at the Cleveland Clinic found that 58.7% of patients reported normal to mild sleep disturbances, and 41.3% reported moderate to severe sleep disturbances (69). In a separate retrospective survey of LC patients, 82.3% reported poor sleep quality (70). Almost half of subjects involved in a study assessing the effects of altered brain perfusion and oxygen levels in LC reported daytime sleepiness, differentiated from LC fatigue (71). Sleep disorders and dysfunction, including non-restorative sleep, are also prevalent in ME/CFS, fibromyalgia, and hEDS/HSD (41, 72–74).

Among patients with recent COVID-19, the frequently reported skin conditions include maculopapular rashes, urticarial lesions, and chilblains (75). LC patients report a range of skin conditions in clinic, however these are not well documented in the literature, thereby making our study one of the first to assess overlapping dermatologic involvement. Skin conditions are also present in hEDS and mast cell activation syndrome (MCAS). In hEDS, skin manifestations include soft skin, hematomas, atrophic scars, piezogenic pedal papules, and cutaneous stretchability (76). Skin manifestations of MCAS include flushing, pruritus, hives, and angioedema, and non-diagnostic skin manifestations include dermographism, urticaria, rashes, edema, alopecia, poor healing, and onychodystrophy (77). More research is needed on the causes, duration, and type of skin lesions in LC.

Our findings also align with research on genitourinary and reproductive health in LC. LC patients have an increased risk for several reproductive health conditions, many of which are also elevated in people with EDS, ME/CFS, or POTS (78). Women with endometriosis had an elevated risk for developing LC in a large EHR study of non-hospitalized LC patients (48). Similarly, multiple studies have found that endometriosis is elevated in women with EDS, ME/CFS, and POTS compared to general population prevalence (78, 79). Women with PCOS in a large population based cohort study had a 28% increased risk for COVID-19 infection after accounting for age and BMI, and PCOS is also elevated in ME/CFS and POTS (78, 80).

The development of autoimmune conditions was also more common after COVID for people diagnosed with LC compared to controls. As of June 2024, following this study’s period of data collection, the presence of autoimmune disorders are included in the definition of LC (4). Autoimmunity has been proposed to play a role in LC even without coexisting diagnosis of conditions that are already established as autoimmune, as suggested by basic science research (81–83). Additionally, two EHR studies of adult Korean and Japanese patients found that COVID-19 is associated with a significant risk for developing autoimmune and autoinflammatory connective tissue disorders (84, 85).

Finally, our study confirms that allergies, sensitivities, and intolerances are common in LC. Allergies and sensitivities to environmental and food antigens are common in ME/CFS, and are included in both the Canadian Consensus Criteria and International Consensus Criteria for diagnosis of ME/CFS (86, 87). In an online survey of Dutch (*n* = 337) and US (*n* = 252) ME/CFS patients, 19.1% of the Dutch cohort and 46.8% of the US cohort reported “sensitivity to smells, food, medications, or chemicals” (88). These new and worsening sensitivities and intolerances have been theorized to be due to mast cell hyperactivation including MCAS (89–91).

### 4.2 Emerging mechanisms

Given the widespread effects of LC on the body, it stands to reason that key underlying mechanisms behind this disease would have the ability to affect multiple organ systems simultaneously. While there is no consensus on the etiology of LC, many theories have emerged (13, 38, 92). Proposed underlying pathologies in addition to immune and neurologic dysregulation have included mast cell disease, endothelial dysfunction and other sequelae of vascular damage, viral reactivation, and mitochondrial impairment, all of which were self-reported as occurring more frequently in people with LC than controls in our study (7, 93, 94). Research groups are increasingly evaluating the role of these pathologies in the context of LC and as related to each other, as well as in other infection-associated chronic conditions (93, 95).

Female sex, and particularly pre-menopausal status, have been associated with LC and conditions of hypermobility, orthostatic intolerance, and ME/CFS (12, 51, 96–99). Low testosterone has also been found to be a risk factor for developing LC (1, 100). Variances in hormonal composition has therefore become an area of interest in understanding the development of infection-associated chronic illness. There are key hormonal as well as chromosomal and metabolic factors that significantly impact both innate and adaptive immune responses to infection, and while such differences can be protective in the context of an acute infection, they may provide a double-edged sword that increases risk for infection-associated chronic illness (101). This may also relate to why a majority of autoimmune diseases, such as Sjogren’s syndrome and systemic erythematous lupus, occur more frequently in females than males, given how autoimmune diseases can themselves be triggered by infections (102–107).

Viral and other microbial infections are strongly linked to the development of ME/CFS, with 70–80% of patients reporting having had a viral infection just prior to the development of symptoms (21–25). Viral infections and viral particles could lead to persistent activation of the innate immune response. Additionally, many viruses are known to localize to mitochondria and utilize mitochondrial machinery for viral replication including SARS-CoV-2, which could lead to mitochondrial dysfunction in numerous cell types and organs (94, 108–116). This corresponds to mitochondrial dysfunction being a hallmark pathology of ME/CFS, and likely LC (38, 93). Oxidative stress, reduced perfusion to tissue, and metabolic dysfunction are additional emerging pathologies that could conceivably contribute to extracellular membrane and connective tissue breakdown (91).

Nervous system involvement is prominent in LC, both as an affected system as well as a mechanism of symptom development. Autonomic dysfunction, observed at higher rates in LC, ME/CFS, and hEDS/HSD, and often manifesting as dysregulated heart rate and blood pressure control, directly contributes to the fatigue, exercise intolerance, and orthostatic symptoms seen in many patients (7, 29, 61, 68, 93, 117). Autoantibodies have also been found in the context of dysautonomia, adding to inflammatory aggravation (82). Small fiber neuropathy commonly coexists with hypermobility and autonomic disorders, contributing to dysthesias and thermodysregulation as well (61). Insular cortex hyperactivation has also been noted across hypermobility, neurodevelopmental conditions such as autism, and dysautonomia, and multiple brain signaling abnormalities have been described in ME/CFS including of the brainstem (93, 118, 119). Advanced neuroimaging has demonstrated altered neurochemical milieu and neuroinflammation in ME/CFS and LC (120–122). Central sensitization syndrome has been proposed as a potential theory behind neurologic symptomatology, but has fallen out of favor given its inability to account for major manifestations such as post-exertional malaise as well as the demonstrated presence of the aformentioned neurologic and immunologic contributors (7, 8, 50, 123, 124).

Hyperactivation of mast cells is a likely mechanism that may link LC, hEDS/HSD, ME/CFS, POTS, and multiple other co-occurring conditions (125–128). Mast cells are antigen presenting cells found throughout the body that activate in response to infections and toxins (129). Mast cells may be activated by SARS-CoV-2 because they express receptors for viral entry, including H1, ACE2, TMPRSS2, NRP1, integrins and cathepsins (126, 127, 130). They then promote proinflammatory T helper (Th)2 responses and Toll-like receptor (TLR)4 induced interleukin (IL)-1β, which has been found to be elevated in LC patients (131–134). A Th2-type immune environment allows viral infections to have an advantage and/or persist, because Th1-type immune responses are needed to clear viral infections (131, 135). Aberrant mast cell activation has also been implicated in exacerbation of autonomic symptoms (136). In particular, mast cells have been found to activate the vagus nerve by release of IL-1β and tumor necrosis factor (TNF) (137, 138). Mast cells are also involved in thromboembolic responses that mediate many poor outcomes in COVID-19 (126, 127). Mast cell hyperactivation is also observed in patients with hEDS/HSD where they respond to defective connective tissue and promote the breakdown of extracellular matrix/connective tissue. Importantly, mast cell numbers and function differ by sex and genetic background, which may partly explain the frequency of LC, ME/CFS, and hEDS/HSD in White females (127, 139, 140).

Finally, tissue laxity worsened by the presence of inflammation may contribute to structural and vascular conditions (53, 56). The inherent connective tissue abnormalities in hEDS/HSD and related conditions can lead to vascular and structural instability. This laxity may impair structural integrity of blood vessels, leading to venous pooling and subsequent orthostatic intolerance. Patients with hEDS and HSD frequently exhibit signs of altered baroreflex sensitivity and increased sympathetic activity, issues that can impair normal autonomic responses to postural and environmental changes (61, 63). Orthostatic flow dysfunction via stasis of venous flow and pressurization, with concomitant venous hypovolemia above the pelvis, has been hypothesized to play a role in patients with pelvic pain secondary to pelvic congestion syndromes (141–143). A newer phenotype along the idiopathic intracranial hypertension spectrum, cerebral venous outflow disorders, was recently described with a high co-occurrence in connective tissue disorders and especially EDS (57). Therefore, it is reasonable to consider the potential contribution of vascular and spinal compression syndromes in LC.

### 4.3 Future research

The multiple intertwined pathophysiologic processes in LC underscores the need for comprehensive research to unravel the precise mechanisms and develop targeted therapeutic strategies. Future research should also explore the frequency of coexisting conditions across the different topics surveyed here, including the order and timeline in which these typically develop, to see if certain diagnoses are more likely to create a risk of or co-develop within LC. Future research should further study mechanisms in LC and similar infection-associated chronic illnesses. Areas could include, but are not limited to, examining the role of breakdown of connective tissue in these illnesses; immune dysfunction (such as mast cell activation), autoimmunity, and chronic inflammation; metabolic and mitochondrial dysfunction; sex and hormonal differences; tissue hypoperfusion; nutrient and mineral depletion; and persistent infections. The true prevalence of the lesser studied pathologies should be better elucidated as well. While our study suggests that potentially critical pathologies such as vascular and endothelial dysfunction, mast cell activation, viral reactivation, and structural/outflow contributors are starting to be discussed with patients, it is likely that involvement of these pathologies are underestimated.

### 4.4 Clinical implications

A strength of our survey is that we simultaneously assessed for numerous potential coexisting conditions. Our findings bring to question the impact of potential underdiagnosis of associated conditions by clinicians. While there are no formal guidelines or approved treatments currently for management of LC, people living with LC may find symptom improvement through management correlating with correctly identified coexisting pathologies and conditions. Long COVID clinics also specializing in connective tissue disorders, ME/CFS, POTS, fibromyalgia, mast cell activation, and associated conditions have effectively applied similar principles to the appropriate LC patients (13, 144, 145).

For example, people with LC identified to have features of post-exertional malaise, often associated with ME/CFS, can benefit from applying a pacing activity management strategy (28, 146, 147). Other non-pharmacologic interventions beneficial for post-exertional malaise, sensory sensitivity, orthostatic intolerance, and other symptoms may also be easily trialed by patients if present (98, 145, 148). People with LC and commonly overlapping illnesses, such as ME/CFS and POTS, should be screened for connective tissue disorders and vice versa. Screening for hypermobile tendencies may open opportunities for management and reduction in joint instability and chronic pain, dysautonomia, and other symptoms.

Similarly, therapeutics employed in overlapping conditions may be harnessed for LC (149). Pharmacologic options utilized in POTS may be helpful in cases of orthostatic intolerance as well as those meeting POTS criteria, as can fibromyalgia medications for pain associated with LC (7, 13, 98). Mast cell therapies and agents that improve endothelial dysfunction have also been associated with symptom improvement in LC (7, 13, 150, 151). Low-dose naltrexone has been helpful in patients with LC, ME/CFS, fibromyalgia, POTS, autism, MCAS, hypermobility, and autoimmune disorders. The beneficial effects of LDN may be secondary to neuroimmune attenuating effects which may further suggest potential overlapping pathologies of these conditions (13, 152–160). Identifying conditions or syndromes with autoimmune associations that could follow COVID-19 infection can allow for informed trials of immunomodulatory therapy (144, 161–166). The role of antimicrobials in managing infection-associated chronic illness is under investigation (144). Identification of structural contributors that are amenable to intervention may alleviate symptoms in select cases (56, 141, 142, 167).

Clinicians suspecting LC should apply the current ICD-10 code, U09.9—Post COVID-19 condition along with the code M35.89 for “other specified systemic involvement of connective tissue” related to COVID-19 if applicable (168, 169). Accordingly, diagnostic codes for overlapping conditions as mentioned here, such as ME/CFS (G93.32) and OI/POTS (G90.A) should also be considered. Additional diagnostic codes are being developed for coexisting pathologies. Use of these codes can improve electronic capture, thereby allowing for more accurate research and management related to these groups.

### 4.5 Limitations

Our study has several limitations. Both study sites were part of a single tertiary/quaternary care center and as such the findings from this cohort of patients may not represent all patient populations. The disparity in representation is also evident in that 93.9% of LC respondents were White. Controls were based only in Minnesota while LC patients were seen at both Minnesota and Florida sites and could be from any location, and there were no responses from non-White individuals for the control group. Furthermore, the control group utilized for this study was limited in number and, consequently, the study had uneven sample sizes. It is possible that severe symptoms affected the ability of some LC patients to participate in the call for survey responses. All participants in this study were at least 3 months from a COVID infection; however, the specific length of LC and whether the patient had multiple infections were not recorded. Although our survey population was aged similar to other studies at an average of 52 years, this could lead to a lower percentage of GJH being reported as GJH reduces dramatically post-menopause. The primarily female sample reflects the female predominance in infection-associated chronic illness groups, however a larger, more balanced sample would be better powered to assess sex differences within systems.

This is a survey-based study which is inherently limited by the recollection of participants regarding symptoms and diagnoses. It is important to acknowledge however that a number of the conditions evaluated in this survey are also only recently being recognized by many infection-associated chronic illness experts, and as such likely underdiagnosed even in our study population. As such, respondents who were evaluated more recently may have been more likely to receive diagnoses based on growing knowledge and recognition of certain comorbidities, compared to those evaluated much earlier in the rise of LC. The screening instruments incorporated into the survey are also insufficient to independently establish a formal diagnosis. Although inclusion of EHR data may have offered additional information, EHR data are especially fallible in the context of new and emerging diseases that may not have associated ICD coding yet.

## 5 Conclusion

In this study, we identified that a number of symptoms and comorbidities are new or worse following SARS-CoV-2 infection in patients diagnosed with LC compared to controls. The LC patients in this study also had similar co-occurrence of ME/CFS, GJH and orthostatic intolerance as other studies. Our paper provides a foundation to examine these relationships further. These findings highlight the presence of multiple underrecognized conditions in the context of LC, especially those involving autonomic dysfunction, connective tissue compromise and immune dysregulation. As the prevalence of different pathologies and conditions is better characterized in LC, research can further elucidate mechanisms that offer the potential for more effective treatments.

## Software

Study data were collected and managed using REDCap electronic data capture tools hosted at Mayo Clinic. 1, 2 REDCap (Research Electronic Data Capture) is a secure, web-based software platform designed to support data capture for research studies, providing (1) an intuitive interface for validated data capture; (2) audit trails for tracking data manipulation and export procedures; (3) automated export procedures for seamless data downloads to common statistical packages; and (4) procedures for data integration and interoperability with external sources (170, 171).

## Data Availability

The datasets presented in this article are not readily available because requests for raw data must be approved by the Mayo Clinic IRB and require a data transfer agreement by standard protocol. Requests to access the datasets should be directed to Stephanie Grach, grach.stephanie@mayo.edu.
