# Supplementary material for: Overlapping conditions in Long COVID at a multisite academic center
Source: Front Neurol. 2024 Oct 25;15:1482917. doi: 10.3389/fneur.2024.1482917 (PMC11543549; doi:10.3389/fneur.2024.1482917)
Supplement: Supplementary file 1 [file Table_1.DOCX]

**Supplemental Table 1. Modified COMPASS-31 questions for orthostatic intolerance.**

| **Question** | **Answer Choices** |
| --- | --- |
| Before COVID, did you felt faint, dizzy, "goofy," or difficulty thinking soon after standing up from a sitting or lying position? | No, Yes |
| When standing up, how frequently would you get these feelings or symptoms? | Rarely, Occasionally, Frequently, Almost Always |
| How would you rate the severity of these feelings or symptoms? | Mild, Moderate, Severe |
| After COVID, have these feelings or symptoms that you have experienced: | Gotten much worse, Gotten somewhat worse, Stayed about the same, Gotten somewhat better, Gotten much better |
| After COVID, do you felt faint, dizzy, "goofy," or difficulty thinking soon after standing up from a sitting or lying position? | No, Yes |
| When standing up, how frequently do you get these feelings or symptoms? | Rarely, Occasionally, Frequently, Almost Always |
| How would you rate the severity of these feelings or symptoms? | Mild, Moderate, Severe |
