# Supplementary material for: Overlapping conditions in Long COVID at a multisite academic center
Source: Front Neurol. 2024 Oct 25;15:1482917. doi: 10.3389/fneur.2024.1482917 (PMC11543549; doi:10.3389/fneur.2024.1482917)
Supplement: Supplementary file 2 [file Table_2.docx]

**Supplemental Table 2. Summary of new or worsening concerns after COVID-19 by sex among Long COVID patients.**

|  | Female (*n*, %) | Male (*n*, %) | Total (*n*, %) | P value |  |
| --- | --- | --- | --- | --- | --- |
| Did your pain condition(s) start or worsen after COVID? | 127 (93.4%) | 41 (97.6%) | 168 (94.4%) | 0.25 |  |
| Did any of your neurologic condition(s) start or worsen after COVID? | 153 (96.2%) | 41 (80.4%) | 194 (92.4%) | 0.001 |  |
| Did your sleep condition(s) start or worsen after COVID? | | 104 (81.9%) | 40 (85.1%) | 144 (82.8%) | 0.33 |
| Did your skin condition(s) start or worsen after COVID? | | 79 (73.1%) | 10 (50.0%) | 89 (69.5%) | 0.093 |
| Did at least one genitourinary condition start or worsen after COVID? | | 58 (60.4%) | 8 (61.5%) | 66 (60.6%) | 1.00 |
| Allergies, sensitivities/ intolerances | |  |  |  | 0.28 |
| *Allergies, sensitivities/ intolerances stayed the same | | 45 (24.9%) | 23 (38.3%) | 68 (28.2%) |  |
| *Allergies, sensitivities/ intolerances new after COVID | | 43 (23.8%) | 12 (20.0%) | 55 (22.8%) |  |
| *Allergies, sensitivities/ intolerances before COVID got worse | | 61 (33.7%) | 16 (26.7%) | 77 (32.0%) |  |
| Diagnosis of a mood disorder (e.g., depression, anxiety) after COVID | | 58 (31.2%) | 20 (33.3%) | 78 (31.7%) | 0.65 |
| Diagnosis of a gastrointestinal disorder (e.g., inflammatory bowel syndrome) after COVID | 44 (23.7%) | 10 (16.7%) |  | 0.29 |  |
| Have you been diagnosed with an autoimmune disorder? |  |  |  | 0.18 |  |
| *before COVID | 28 (16.1%) | 6 (10.2%) | 34 (14.6%) |  |  |
| *after COVID | 32 (18.4%) | 6 (10.2%) | 38 (16.3%) |  |  |
| *before + more after COVID | 3 (1.7%) | 2 (3.4%) | 5 (2.1%) |  |  |
| Diagnosis of a pulmonary disorder (e.g., asthma, interstitial lung disease) after COVID | 30 (16.1%) | 9 (15.0%) | 39 (15.9%) | 1.00 |  |
| Diagnosis of a cardiac disorder (e.g., myocarditis, heart failure) after COVID | 24 (12.9%) | 6 (10.0%) | 30 (12.2%) | 0.65 |  |
